# Clinical efficacy and safety of methotrexate compared with leflunomide in the treatment of rheumatoid arthritis: A protocol for systematic review and meta-analysis

**DOI:** 10.1097/MD.0000000000028285

**Published:** 2021-12-23

**Authors:** Weiyu Qi, Yu Xia, Xin Li, Jianzhong Cao

**Affiliations:** a Hunan University of Chinese Medicine, Changsha, China; b Hunan Provincial Key Laboratory of Diagnostics in Chinese Medicine, Hunan University of Chinese Medicine, Changsha, China.

**Keywords:** leflunomide, meta-analysis, methotrexate, rheumatoid arthritis

## Abstract

**Background:**

Methotrexate and leflunomide are classic treatments for rheumatoid arthritis (RA), however, which is the best choice for patients of RA is still an important question clinically, and this meta-analysis is used to systematically evaluate and compare their efficacy and safety.

**Methods:**

We searched PubMed, Cochrance Library, Embase, SinoMed, China National Knowledge Infrastructure, China Science and Technology Journal Database, WanFang Data databases. The retrieval time was from the establishment to September 7, 2021. Literature screening, data extraction, and quality assessment were performed according to the Cochrane risk of bias tool. Meta-analysis of the included studies was performed using RevMan 5.3 software and Stata 12.0 software.

**Results:**

The clinical efficacy and safety of leflunomide and methotrexate are evaluated by American College of Rheumatology (ACR)20/50/70, DAS28, total effective rate, adverse reaction rate, morning stiffness, swollen joint count, tender joint count, erythrocyte sedimentation rate, C-reactive protein, and rheumatoid factor.

**Conclusion:**

The results of this meta-analysis will provide reliable evidence clinical efficacy and safety for RA. More high-quality randomized controlled trials are still needed to provide more reliable evidence for the treatment of RA.

**PROSPERO number:**

CRD42021270980

## 1 Introduction

Rheumatoid arthritis (RA) is an autoimmune disease characterized by progressive synovitis, pannus neoplasia,^[1]^ articular cartilage erosion, and bone destruction.^[2]^ The incidence rate of RA in China is about 0.45% to 1.0%, and the disability rate in 5 years is as high as 43.5%, which could be aggravated with the prolongation of the disease course.^[3]^ Both the European League Against Rheumatism recommendations (updated in 2019) and the 2018 Chinese guidelines for the treatment of RA clearly indicate that traditional synthetic disease-modifying antirheumatic drugs are the cornerstone drugs for the treatment of RA.^[4,5]^ Methotrexate (MTX) and leflunomide (LEF) are recognized as the first choice disease-modifying antirheumatic drugs for the treatment of RA at home and abroad,^[4,5]^ with definite clinical efficacy.

However, MTX and LEF have certain hepatotoxicity^[6]^ and many other adverse reactions, such as decreased white blood cells and diarrhea.^[7]^ Therefore, we use meta-analysis to compare the effectiveness and safety of these 2 drugs for a better therapeutic method of RA.

## 2 Materials and methods

### 2.1 Ethics statement

All analyses were based on previously published studies, this article does not contain any studies with human participants or animals performed by any of the authors, thus ethical approval and patient consent are not applicable.

### 2.2 Search strategy and selection criteria

This meta-analysis was conducted according to the guidelines of the Preferred Reporting Items for Systematic Reviews and Meta-Analyses statement. Chinese databases such as China National Knowledge Infrastructure, WanFang, China Science and Technology Journal Database, and English databases such as PubMed, Cochrane Library were searched. The retrieval time was from the establishment to September 7, 2021. The search terms were included: “rheumatoid arthritis” OR “RA” AND “methotrexate” OR “MTX” AND “leflunomide” OR “LEF” AND “clinical trial” OR “randomized controlled trial.”

The inclusion criteria were as follows:

(1) **P**articipants: RA patients, and the diagnostic criteria referred to the 1987 American College of Rheumatology (ACR) diagnostic criteria for RA^[8]^;
(2) **I**nterventions: The experimental group was treated with MTX;
(3) **C**omparators: the control group was treated with LEF;
(4) **O**utcomes: The primary outcomes were ACR20/50/70, DAS28, total effective rate, and adverse reaction rate; the secondary outcomes were morning stiffness, swollen joint count, tender joint count, erythrocyte sedimentation rate, C-reactive protein, and rheumatoid factor;

The exclusion criteria were as follows:

(1) Repeated published studies;
(2) The data is incomplete, the outcome effect is not clear, and the data cannot be extracted for analysis;
(3) Animal experiments, cell experiments and the review literature;
(4) Clinical case reports;
(5) The control group was combined with other treatments.

### 2.3 Data extraction and quality assessment

Two researchers independently performed the data extraction and quality assessment according to the screening criteria, and cross-checked them. If there are conflicts of opinions, resolve them through collective discussion in the research team. In the process of literature screening, the literature with irrelevant titles were excluded, and the abstracts and full texts should be further read to determine the final included literature. Basic information was extracted from the included studies (first author, year of publication, number of patients, interventions, and course of treatment). The primary outcomes were ACR20/50/70, DAS28, total effective rate, and adverse reaction rate; the secondary outcomes were morning stiffness, swollen joint count, tender joint count, erythrocyte sedimentation rate, C-reactive protein, and rheumatoid factor. The quality of the included studies was evaluated according to the Cochrane Risk of Bias tool.^[9]^

### 2.4 Statistical analysis

RevMan 5.3 software and Stata 12.0 software was used for meta-analysis. Odds ratio was calculated for the dichotomous variable, mean difference was calculated for the continuous variable. All of them were expressed with 95% confidence interval. *I*^*2*^ and chi-square tests were used to assess the heterogeneity. The results were shown in the forest plot. For all outcomes, Egger and Begg test were used to detect publication bias.

## 3 Results

### 3.1 Literature search results

**Figure 1:**
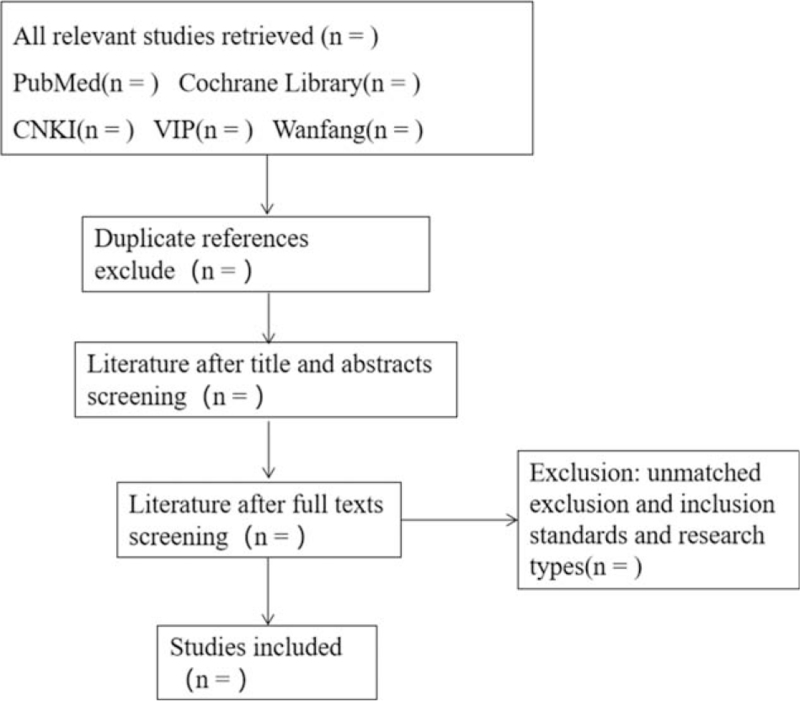
Flow chart of study selection.

The screening process is shown in Figure 1.

### 3.2 Publication bias analysis

Begg and Egger test are used to evaluated the publication bias analysis of this study.

### 3.3 Grading the quality of evidence

The Grading of Recommendation, Assessment, Development, and Evaluation system will be used to appraise the quality of evidence from the studies obtained. The levels of it will be divided into high, moderate, low, very low.

## 4 Discussion

Studies have shown that MTX is an inhibitor of folate reductase, and its mechanisms in the treatment of RA are as follows:

(1) It mainly inhibits the reduction of dihydrofolate reductase into tetrahydrofolate, which blocks the transfer of -C group during the biosynthesis of purine nucleotides and pyrimidine nucleotides, inhibits DNA synthesis, and then inhibits the proliferation of synovial cells.^[10]^
(2) Regulating the balance of Th1/Th2 and Th17/Treg, thus to inhibit chronic synovitis.^[11,12]^
(3) Regulating adenosine-related pathways and inhibiting synovitis of RA.^[13]^
(4) Inhibiting the expression of MMP-1, MMP-2, and other MMPs, so as to inhibit the destruction of joint bone.^[13]^
(5) MTX can inhibit bone destruction by upregulating serum OPG, decreasing the expression of RANKL, competitively inhibiting the binding of RANK and RANKL, and regulating the RANKL/RANK/OPG pathway.^[14]^

The mechanisms of LEF in the treatment of RA are as follows:

(1) The metabolite A771726 inhibits the synthesis of pyrimidines by inhibiting mitochondrial dihydrofolate dehydrogenase, then leading to the proliferation of T cells.^[15]^
(2) A771726 inhibits the infiltration of synovitis by inhibiting p38 MAPK pathway.^[16]^
(3) Inhibiting bone erosion by acting on AHR-CRP signal.^[17]^
(4) Inhibiting the expression of MMP-1, MMP-9, and other MMPs, thereby inhibiting the destruction of joint bone.^[18,19]^ It can be seen that MTX and LEF work through different targets to improve clinical symptoms.

Pharmacokinetic studies have found that after oral administration of MTX, the absorption rate is fast with a half-life of 7 to 10 hours, and a large amount of MTX accumulates in the liver. The continuous use of a small dose may cause the elevation of alanine aminotransferase, which leads to drug-induced liver damage.^[20]^ Oral administration of LEF also has a fast absorption rate and it changes to A771726 in the liver. Continuous use of LEF may cause elevation of alanine aminotransferase, thus leading to drug-induced liver damage.^[21]^ LEF is cheaper than MTX, so it is considered that LEF is more worthy of being adopted in the clinical treatment of RA.

It is suggested that RCTs should be conducted with the following considerations in the future:

(1) Invite experts of RCTs methodology and evidence-based medicine to participate in the design of standardized and rigorous high-quality RCTs research protocols;
(2) Register in the Chinese Clinical Trial Registry or ClinicalTrials.gov prior to implementation of the RCTs.

## Author contributions

**Data curation:** Weiyu Qi, Yu Xia.

**Software:** Weiyu Qi, Yu Xia.

**Supervision:** Xin Li.

**Writing – original draft:** Weiyu Qi.

**Writing – review & editing:** Jian-Zhong Cao.
